# Associations between Learning and Behavioral Difficulties in Second-Grade Children

**DOI:** 10.3390/children7090112

**Published:** 2020-08-26

**Authors:** Emanuela Castro, Maria Cotov, Paola Brovedani, Gabrielle Coppola, Tania Meoni, Marina Papini, Tania Terlizzi, Chiara Vernucci, Chiara Pecini, Pietro Muratori

**Affiliations:** 1 IRCCS Fondazione Stella Maris, Scientific Institute of Child Neurology and Psychiatry Pisa, 56018 Calambrone, Italy; maria.cotov@gmail.com (M.C.); paola.brovedani@fsm.unipi.it (P.B.); marina.papini@outlook.it (M.P.); chiara.ver@gmail.com (C.V.); pmuratori@fsm.unipi.it (P.M.); 2 Department of Education, Psychology, Communication, University of Bari, 70121 Bari, Italy; gabrielle.coppola@uniba.it; 3 CRED Della Zona Educativa Pisana, 56121 Pisa, Italy; dsa.zonapisana@gmail.com (T.M.); cred.zonapisana@gmail.com (T.T.); 4 Department of Education, Language, Interculture and Psychology, University of Florence, 50121 Florence, Italy; chiara.pecini@unifi.it

**Keywords:** screening, hyperactivity, emotional problems, mathematical skills, reading skills

## Abstract

Learning and behavioral difficulties often emerge during the first years of primary school and are one of the most important issues of concern for families and schools. The study was aimed at investigating the co-occurrence of difficulties between academic learning and emotional-behavioral control in typically developing school children and the moderating role of sex. A sample of 640 second-grade school children participated in the study. This study used the Strengths and Difficulties Questionnaire to measure the emotional and behavioral difficulties and a battery of objective and standardized tests to evaluate the learning skills in children. In this sample 7% to 16% of children performed below the normal range in reading and/or arithmetic tests. Mixed models showed that children’s hyperactive behaviors were positively related to both reading and math difficulties, and emotional problems correlated negatively with reading accuracy. The more children displayed behavioral difficulties, the more they were exposed to the risk of worsening reading and math performance, especially for girls. The result that among different emotional-behavioral problems within the school setting, hyperactivity behaviors and emotional difficulties are related to learning difficulties with a moderate effect of sex, needs to be taken into account in screening and prevention programs for learning difficulties in order to not disregard the complexity of the associated profiles.

## 1. Introduction

Specific Learning Disorder (SLD) is a broad category within the classification of neurodevelopmental disorders that includes persistent difficulties in reading, writing, arithmetic, or mathematical reasoning skills during the formal years of schooling. Current academic skills, assessed with culturally and linguistically appropriate tests, must be well below the average of the age group. Deficits must not be better explained by developmental, neurological, sensory, or motor disorders and must significantly interfere with academic achievement, occupational performance, or daily living activities [1]. The prevalence of SLD in primary school ranges between 3% and 10%, depending on the country and the inclusion criteria [1,2,3,4,5,6]. In the absence of early and tailored interventions [7,8], SLD can determine negative lifelong effects, such as a higher risk of school drop-out, poorer mental health, and vocational difficulties. Although the diagnosis is made starting from the end of the second grade (7- to 8-year-olds) [9], delays in acquiring basic academic skills in the first primary school years, broadly defined as “learning difficulties”, represent a significant risk for SLD. Thus, screening evaluations are crucial for early identification of the learning difficulties and implementation of preventive interventions for SLD [10].

As for learning disorders, emotional and behavioral difficulties often emerge in the primary school years and are one of the most important reasons for concern for teachers, educators and school administrators. Such difficulties show high stability over time and are associated with long-term negative outcomes, such as school failure and psychopathology [11,12,13].

The co-occurrence of emotional-behavioral and learning difficulties has been documented by several studies in both clinical settings and in typical development, the latter focused on dimensional quantitative traits. Mundy et al. [14] reported, in a community sample of 1239 Australian primary school children, that about one out of five boys and one out of seven girls displayed emotional or behavioral problems (behavioral screening questionnaire), together with significant delays in literacy and mathematic skills. 

Attention-Deficit/Hyperactivity Disorders (ADHD) has been frequently associated with a higher incidence of SLDs in school-age children [15,16,17] and in typically developing children a strong relationship has been reported between hyperactive behaviors and learning difficulties [18,19]. A systematic review [20] supports the notion that attention-deficit/hyperactivity behaviors, both in clinical and non-clinical samples, predict poorer learning abilities and worse academic performance during the entire school period.

Conduct problems also often correlate with learning difficulties [21]. Miles and Stipek [22] analyzed the developmental pathways in two cohorts of 400 low-income English children and found that conduct problems were related to poorer reading abilities in the third and fifth grades, but no relation was found earlier. Wu et al. [23] found that conduct problems were negatively related to mathematical learning skills in a sample of 366 second and third grade US children.

Some studies investigated the relations between internalizing behavioral difficulties and learning difficulties. Mundy et al. [14] found a significant relationship between emotional problems and reading and mathematical abilities while Wu et al. [23] did not confirm a correlation between anxiety symptoms and performance in mathematics. Internalizing symptoms have been systematically reported in developmental dyslexia (for a review, see Mugnaini and colleagues [24]).

Both for SLD and emotional-behavioral disturbances, the impact of the environmental risk variables is still under study (familial socio-economic status, gestational weeks and birth weight, maternal cigarette smoking, family history of psychiatric and medical diseases, and home literacy environment), together with the genetic contributions [25].

Sex has been reported to be a frequent moderating factor in affecting learning skills, confirmed in several studies. Boys have been found to have a higher frequency of learning difficulties with respect to girls in a review of four large epidemiological studies on 7- to 15-year-olds (Rutter et al. [26]). Males performed worse than females in reading accuracy with a greater variability in performance, which increased for lower reading scores in Arnett et al.’s study assessing a sample of 2041 American youths aged 7–24 years [27]. Landerl and Moll [28] reported that reading fluency and spelling skills were separately affected by sex in a population-based sample of 2586 Austrian primary school children. In their study, there was no sex-rate difference in reading speed performance; however, males performed significantly worse in spelling tests than females, pointing to this skill as affected by sex (see also [5]). In an English community sample of 1004 primary school children, Devine et al. [29] evaluated reading and mathematical skills, reporting more males than girls with reading difficulties, but no difference in the sex-rate in subjects with mathematical difficulties. In contrast, Barbaresi et al. [30] reported higher rates of arithmetic difficulties for boys than for girls in an American cohort study on 5718 school-aged children.

Complex relationships probably explain the sex effects linking learning and behavioral difficulties. Willcutt and Pennington [31] indicated that the association between behavioral and learning difficulties in children and adolescents is stronger in boys than in girls. Similarly, in Mundy et al.’s [14] study, only boys showed a significant association between poorer mathematical and reading skills and higher levels of emotional and conduct difficulties. On the contrary, Czamara et al. [18] found that the association between behavioral and learning difficulties was higher in girls than in boys.

Overall, future research is needed on the moderating effects of the children’s sex in determining the relationship between learning and behavioral difficulties.

### This Study

The current study is part of a screening project aimed at providing data on the occurrence of learning difficulties in second-grade children and at investigating the relations between behavioral difficulties, such as hyperactivity, conduct problems, and emotional problems, and learning difficulties in reading and math.

A large population of Italian children attending the second trimester of the second grade was chosen as, for many behavioral and learning disorders, this period represents a watershed between typical, delayed, and impaired scholastic performance. During this time span, when inter-individual variability in learning acquisition is still high and a diagnosis of SLD is not usually confirmed until the end of the second school year, early assessment and intervention are crucial. Moreover, it is acknowledged that emotional-behavioral difficulties in later grades may be secondary to the persistent or aggravation of learning difficulties [22]. Thus, this timeframe may be optimal for analyzing if an association between learning and emotional-behavioral difficulties exists and for characterizing modulating factors.

The study contributes to the existing literature on several aspects, the most important being that academic skills were tested directly with the gold standard measures for identifying children at risk of SLD and emotional-behavior functioning was assessed with validated questionnaires measuring different components. This approach could allow a more detailed analysis of the associations between the two domains and possible mediating factors, specifically the children’s sex. Secondly, but not less importantly, the early and combined assessment of academics and behavior could offer a crucial window of preventive opportunity for targeting both aspects.

A high frequency of children at risk for learning difficulties and a multi-faced pattern of associations between difficulties in academic learning and emotional-behavioral control were expected [14,15,16,17,18,19,20,21,22,23,24]. Nevertheless, how different academic skills are associated to different emotional-behavioral problems and how the child’s gender may moderate the relations between behavioral and learning difficulties were investigated without a priori hypotheses, given that the evidence on such associations and moderating factors is still controversial [14,18,31].

## 2. Materials and Methods

### 2.1. Participants and Procedures

Participants were recruited from the 42 second-grade classrooms of the 27 primary schools in the districts of Pisa (Italy) for a total of 750 eligible participants. Information letters explaining the aims of the study were sent to all parents of children in the participating schools. Parents or legal guardians gave their written consent to allow children to participate in the study. Of all children invited to participate, 91% were enrolled in the study and were all regularly attending the second grade. The sample comprised 680 students (366 males) divided into 42 classes (mean age = 7.24 years, sd = 0.45 years; range = 6–8 years). Data from children with a neurodevelopmental disorder diagnosis (Intellectual Disabilities, Communication Disorders, Autism Spectrum Disorder and Motor Disorders; n = 40), identified on the basis of their medical certifications and of the teachers’ reports, were excluded from the analysis. Teachers involved in the project (n = 42) had a mean age of 42.25 years (sd = 2.32 years) and eight years of teaching experience on average. During the month of December 2018, all teachers were trained, with a six-hour workshop delivered by four school psychologists, on the use of screening measures to detect learning and behavioral difficulties. Clear written instructions handed out to all teachers and continuous supervision meetings were also provided. During the following month (January 2019), teachers assessed children with reading and arithmetic tests and filled out the behavioral questionnaire described below. The four school psychologists involved in the project scored the tests and the questionnaire. The number of individuals who completed each assessment is reported at the beginning of the results section. The study was conducted in accordance with the Declaration of Helsinki, and the protocol was approved by the Ethics Committee of each school (PTOF 2018–2019). The “CRED” (Centro Ricerca Educativa Didattica) Institutional Board approved all the procedures performed in the study.

### 2.2. Measures

For the evaluation of behavioral difficulties, teachers filled out the Strengths and Difficulties Questionnaire (SDQ) [32], the validated Italian version [33]. This is a 25-item questionnaire, for children between four and 16 years, evaluating the presence of behavioral problems on four scales: (1) conduct problems (e.g., bullying); (2) hyperactivity (e.g., squirming); (3) emotional symptoms (e.g., worrying); (4) peer problems (e.g., not liked by other children). Each item is ranked on a Likert-type scale from zero to two; each scale score can range from zero (absence of difficulties) to 10. The SDQ includes a scale evaluating the presence of prosocial behaviors, which was not used in this study. In the sample, the SDQ reliability was generally satisfactory, as demonstrated by the following mean internal consistence for each subscale (Cronbach’s α): 0.73 for conduct problems, 0.77 for hyperactivity, 0.81 for emotional symptoms and 0.80 for peer problems.

For assessing the text reading skills, The MT reading battery for primary school (see [34] for psychometric properties) was utilized. It is a standardized national reading test providing three measures: the speed and accuracy of decoding and reading comprehension. Raw scores (syllables per second and number of errors, for reading speed; correct responses for text comprehension) fall into four categories: High Normal, Average, Borderline and Impaired. Reading accuracy and speed were measured individually. Each child, seated alone in a quiet room, was instructed to read aloud the passage as accurately as possible at his/her own pace while teachers recorded time (seconds) and number of errors. The maximum duration of the test is four minutes, after which it is interrupted. Reading comprehension was measured collectively. Children had to silently read the passage without time limits and answer 10 multiple-choice questions with four options. For reading speed and comprehension, a higher score indicates better performance; for reading accuracy, a higher score indicates worse performance.

For mathematical skills, the arithmetic sub-tests of the standardized screening battery (AC-MT 6–11) was administered (for psychometric properties see [35]). Raw scores are translated into four performance categories: High Normal, Average, Borderline and Impaired. Because of the speed–accuracy trade-off (e.g., fast answers tend to be less accurate or vice versa), in the present study the two measures were aggregated through a Principal Axis Factoring. The two variables loaded on one factor, accounting for 68.02% of the variance. Both measures correlated positively with the factor scores, suggesting that children with higher scores committed more errors and were slower in their mental arithmetic performance. The factor was labeled as mental arithmetic difficulties and it was used for statistical analyses. Children were tested individually. Teachers read aloud each of six additions/subtractions and asked the child to mentally calculate the result. Time to complete each calculation and the number of errors were recorded. 

The order of the mathematics and reading assessments was randomized.

### 2.3. Statistical Analysis

The data analyses were run using the Statistical Package for the Social Sciences (SPSS) version 24 (IBM Corp, Armonk, NY, USA). Preliminary analyses consisted of calculating the number of cases falling below the borderline cut-off as a function of sex. Sex differences were then explored across the outcome measures of interest. The associations between the predictors and outcomes were tested using multilevel models, which allow treating non-independent measures, given the hierarchical data structure, with children (Level 1) nested within classrooms (Level 2). Prior to conducting multilevel analyses, intra-class correlations (ICC) were calculated for each outcome variable by fitting a fully unconditional model (i.e., Model 0 with only a random intercept and with no predictors). The ICC indicates if there is a significant amount of variability in the outcome explained by the nesting or contextual variable (i.e., classroom). Models predicting each outcome included the fixed effects of sex, conduct problems, hyperactivity, difficulties with peers and emotional problems. Moreover, the interaction terms between each of these emotional-behavioral difficulties and sex were also entered in order to explore whether sex moderated the impact of the indicators of emotional-behavioral functioning on each outcome, by increasing or buffering their effects. The predictors were all standardized into z-scores and since some violated normality, all were transformed into normal distributions through the Van der Waerden formula. The between-classroom variance was estimated by entering the intercepts for classrooms as the random effects.

## 3. Results

Out of the total sample (680 children), 640 completed the reading comprehension test with 11.8% (77 children, 40 males) scoring below the borderline cut-off. No significant sex difference was found in this group (χ^2^ = 5.56, *p* = 0.47). For reading speed, of the 620 children who completed the test, 13.1% (81 children, 36 males) had scores falling within the borderline category and neither in this group was there a significant difference in sex rate (χ^2^ = 7.36, *p* = 0.07). For reading accuracy, of the same 620 children, 15.9% (99 children, 49 males) performed below the borderline cut-off, without significant differences in sex rate (χ^2^ = 2.93, *p* = 0.40). Among the 640 children who completed the mental arithmetic test, 7.2% (46 children, 22 males) performed below the borderline cut-off. No significant sex rate difference was found in this group (χ^2^ = 2.94, *p* = 0.56).

For the Strengths and Difficulties Questionnaire, preliminary analyses consisted of correlations among the four indicators of behavioral difficulties (data available for 580 children). Pearson’s r correlations ranged from 0.29 and 0.65 and are reported in Table 1. Tabachnick and Fidell’s criteria [36] (p. 87) was applied, according to which multicollinearity is proven when the conditioning index is greater than 30 and at least two variance proportions are higher than 0.50 for a given root number. The data did not satisfy these conditions (e.g., all conditioning indices were below 2.5), thus excluding the collinearity effects in the results. Additionally, the variance inflation factor (VIF) was calculated for each predictor: the VIF represents the proportion of variance in one predictor explained by all the other predictors in the model. Ranges of the VIF values for each SDQ dimension were as follows: emotional difficulties: 1.97–1.44; conduct problems: 1.38–1.26; hyperactivity: 1.52–1.21; and difficulties with peers: 1.86–1.17. Given that all values were below the suggested cut-off of 2, multicollinearity could be excluded [37].

In testing associations between learning and behavioral difficulties, Model 0 with only a random intercept showed that the variation across classrooms accounted for a significant proportion of the variance in all but two outcomes. For the text reading parameters, ICC were 0.16, 0.05 and 0.02, respectively, for accuracy (indexed as errors), speed and comprehension, suggesting that 16%, 5% and 2% of the variance of each of these outcomes was explained by belonging to the same classroom. The amount of variance explained by the nesting variable was significant for reading accuracy (Z Wald = 2.79, *p* < 0.001), marginally significant for reading comprehension (Z Wald = 1.79, *p* = 0.07) and did not reach significance for reading speed (Z Wald = 0.95, *p* = 0.30). For all three outcomes, the amount of unexplained variance was significant (16.98 < Z Wald < 17.18, *p* < 0.001). For the mental arithmetic skills, ICC was 0.07 (Z Wald = 2.03, *p* < 0.05), suggesting that also for this outcome a significant part of the variance depended on the nesting variable. Nevertheless, also for the arithmetic outcomes, the amount of unexplained variance was significant (Z Wald = 16.56, *p* < 0.001). The results from the decomposition of variance legitimated using multilevel models for almost all of the outcomes and for all of them the significance of the unexplained variance recommended testing further models with predictors.

Sex differences on the study’s measures were explored with independent t-tests. Results, reported in Table 2, show that males, compared to females, had a significantly greater number of conduct problems, hyperactivity and difficulties with peers. While no sex differences emerged on the three reading measures, girls were faster than boys in mental arithmetic calculation, with comparable accuracy.

Mixed models predicting each outcome are reported in Table 3, Table 4, Table 5 and Table 6. These models reveal that, among the indicators of emotional-behavioral difficulties, behaviors related to hyperactivity were the ones interfering the most with children’s learning, having a negative impact on reading comprehension, speed and accuracy, as well as on mental arithmetic. Moreover, being male is related to a better performance in reading comprehension, speed and accuracy, as well as in arithmetic computations. As to the other main effects, emotional difficulties are related to a lower reading accuracy and lower reading comprehension.

**Figure 1:**
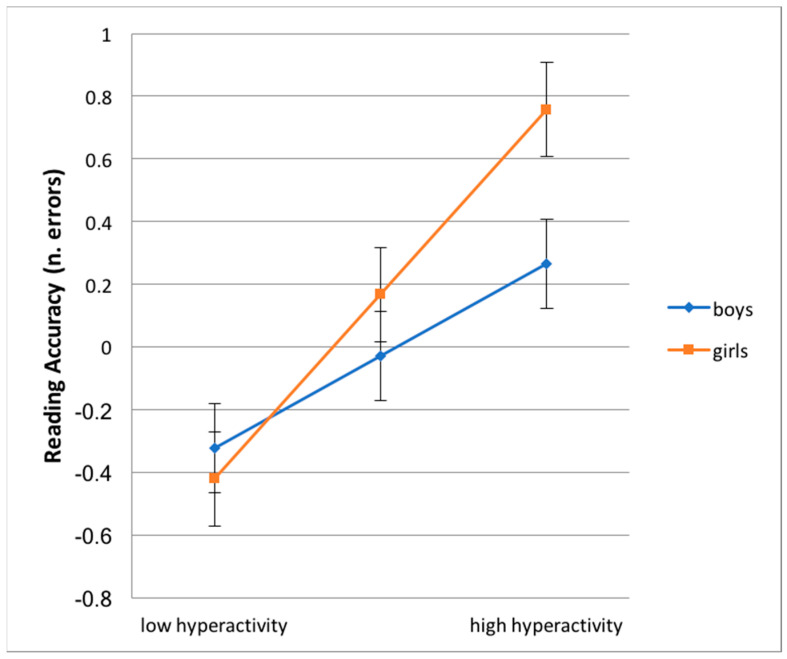
Interaction hyperactivity (x-axis) by sex in the prediction of reading accuracy (no. of errors, y-axis). Note: Error bars show the 95% confidence interval (CI) for the regression coefficients.

**Figure 2:**
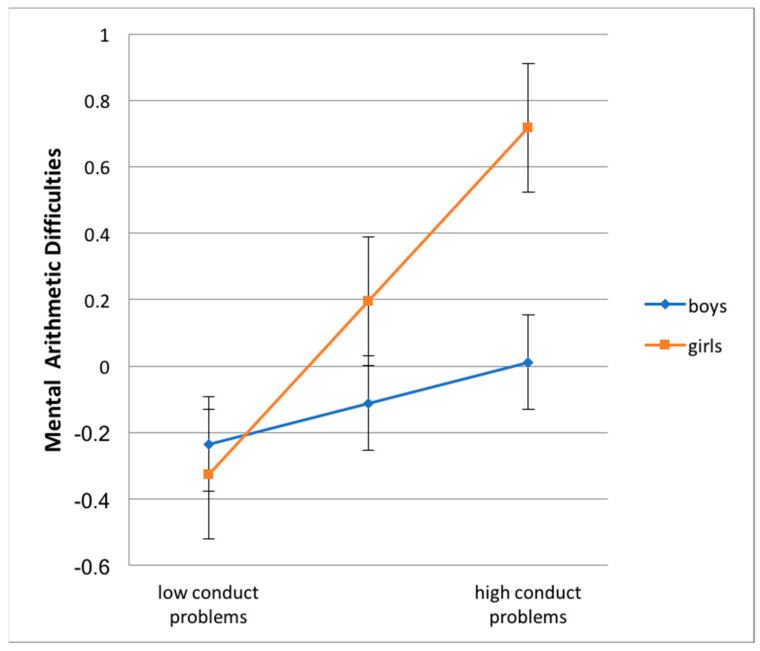
Interaction conduct problems (x-axis) by sex in the prediction of mental arithmetic difficulties (y-axis). Note: Error bars show the 95% confidence interval (CI) for the regression coefficients.

In order to explore the interaction effects (Table 7 and Table 8), the mixed models were re-run separately for each sub-group of children divided by sex [38]. Results show that the levels of hyperactive behaviors were related to worse performance in reading accuracy, both for males and for females, with such a relation being much stronger for the latter (b = 0.293, *p* < 0.001, intercept = −0.029, n.s., for boys; and b = 0.589, *p* < 0.001, intercept = 0.168, n.s., for girls,). This result suggests that the more the child is hyperactive the more he/she is exposed to the risk of a worsening reading performance, especially for females (see also Figure 1). Sex interacted significantly with conduct problems in the prediction of mental arithmetic difficulties: the single slope analysis in Table 8 shows that conduct problems lost predictive power for boys, b = 0.123, n.s., intercept = −0.112, n.s., while remaining significant among the sub-group of girls, b = 0.521, *p* < 0.001, intercept = 0.196, n.s. By focusing attention on both the moderating effect and the comparison between the intercept values of the two groups, the overall results suggest that females, compared to males, have on average much more difficulties in mental arithmetic and that such a risk increases consistently if they also display conduct problems (see also Figure 2).

## 4. Discussion

This study was aimed at providing the frequency of learning and behavioral difficulties in a large sample of typically developing second-grade Italian children and at investigating the associations between them. Learning difficulties in reading comprehension, speed and accuracy were directly evaluated with the gold standard tests for clinical settings while behavioral problems were assessed using a standardized questionnaire filled out by the teachers. For reading speed, 13.1% of the children had borderline scores, while for reading accuracy, 15.9% scored below the borderline cut-off. In the mental arithmetic test, 7.2% performed below the borderline cut-off. A substantial number of second graders thus displayed below average to impaired performance in reading and mental arithmetic.

As expected, the occurrence of learning difficulties was higher than the prevalence of SLD reported by international and national guidelines (3–10% in primary school-aged children) [3,4,5,6] and by the Italian Ministry of Education (around 2–6% of the Italian population in the 2017–2018 school year). The high incidence of children scoring significantly below the population mean at the reading and mental arithmetic tests should be interpreted with caution. This percentage of at risk-children probably includes children who will develop SLD but also those who will resolve learning delays spontaneously, and those children whose learning difficulties are secondary to other problems. However, the current findings indicate that the second-grade period represents a critical time window for early identification of SLD risk and for understanding the complex interplay between learning and emotional-behavior difficulties, prompting greater attention to prevention and early treatment options within the school system.

Indeed, a further relevant finding of the study was the expected association between emotional-behavioral problems and learning difficulties. In particular, not conduct nor peer difficulties but rather children’s hyperactive behaviors and emotional problems were related to difficulties in reading comprehension, speed and accuracy, as well as in arithmetic. These findings confirm previous studies indicating a strong relation between hyperactivity and reading decoding difficulties [18,20,30,39] and extends them by showing the effect of hyperactivity also on reading comprehension. Because of the cross-sectional nature of our data, no conclusion can be drawn on whether there is a causative relationship between the hyperactive behaviors and reading difficulties. Nevertheless, previous studies supported the hypothesis that hyperactivity and reading might share genetic, environmental and neuropsychological risk factors [16,40]. Documenting this association in a typical population before clinical diagnoses can be formulated appears extremely relevant for early prevention/intervention given that reading difficulties and hyperactive behaviors may mutually reinforce each other [41]. The literature on the relationship between hyperactive behaviors and math difficulties is less consistent. Monuteaux et al. [42] suggested that math difficulties are independent of hyperactivity behaviors. However, hyperactivity behaviors were strongly related to reading difficulties as well as to math difficulties in a large community sample of Australian children [14]. The results of the present study suggest a strong relationship between hyperactivity behaviors and math difficulties, a putative indicator of subsequent developmental dyscalculia.

Another main finding was the association between the levels of children’s emotional problems and their performance in reading accuracy. Specifically, greater emotional problems were related to worse performance in this task. This is a novel finding, albeit only limited to reading accuracy, which needs further evidence. In agreement with Grills-Taquechel et al. [43], one can assume that higher levels of emotional problems may disrupt children’s ability to focus on the learning tasks, which could further increase their anxiety-related behaviors and worsen their performance.

Concerning the sex ratio of children at risk for SLD, no significant differences were found for children scoring below the borderline cut-off for reading comprehension, reading speed, reading accuracy and mental arithmetic. To our knowledge, this lack of a sex effect in reading performance is novel evidence [26,27] and not expected. For arithmetic skills, instead, the results are consistent with previous studies, which did not find differences in the sex rate of at-risk subjects [8,29]. Concerning sex as a moderator, girls with hyperactivity performed worse on reading tests than males and girls with conduct problems performed worse on the arithmetic test than males. These results suggest that females with higher levels of behavioral difficulties are particularly at risk for impaired scholastic performance. In agreement with Mano et al. [44], our findings indicate that the relation between behavioral and learning difficulties seems to be stronger for girls and such a risk increases consistently if they also present more severe externalizing problems. This is consistent also with Czamara et al.’s findings [18], that higher hyperactivity SDQ-scores predicted greater literacy difficulties, this relation being stronger for girls. Displaying one of the two conditions placed females at higher risk of having the other one as well. Although the data in the present study do not allow causal interpretations, one can assume that coping strategies for emotional problems may differ between boys and girls and differentially affect school performance.

### Limitations

There are a number of limitations that could have reduced the strength of the results. The level of intelligence, not directly evaluated, could have mediated the associations between learning and emotional-behavioral skills. However, behavioral and genetic studies suggest that relations between learning and behavioral difficulties are likely to be not directly dependent on IQ (see, for instance, [45]). Another limitation is that the children’s behavioral difficulties were rated exclusively by teachers. Even if teachers were closely trained and supervised on test administration, bias could have influenced the results. Moreover, data on families’ socio-economic status was not controlled for as a risk factor for learning difficulties [25]. However, the large number of schools participating in the study may have had a leveling effect of this possible moderating factor.

Finally, only the SDQ emotional symptoms (e.g., worrying) subscale was used to assess the children’s emotional distress. Future studies could consider broader and more specific measures of emotional development and behavioral control.

## 5. Conclusions

The findings from this study are relevant in terms of the relations between learning difficulties and hyperactivity behaviors and emotional problems in the second-grade school context and cannot be extended to other behavioral-emotional difficulties. However, this study has important implications for the school psychology field. In Italy, school psychologists offer screening assessments and/or a preventions intervention either for learning difficulties or for behavioral-emotional issues in a school setting. This study suggests that behavioral difficulties in school may be strongly related to learning difficulties, and thus a combined assessment of both difficulties is crucial.

While the evidence for specific intervention strategies to reduce either the levels of children’s behavioral difficulties, or the levels of children’s learning difficulties is convergent, the most appropriate intervention strategy for children who have high levels of both behavioral and learning difficulties is unclear [46,47,48,49]. There is some evidence that providing social-emotional learning intervention could impact both behavioral problems and learning difficulties [46,47]; however, existing empirical evidence addressing this issue is sparse.

There are two other important implications for school psychologists stemming from the results of this study. First, they suggest that, alongside hyperactivity behaviors, emotional problems also could be related to the levels of children’s reading difficulties. Secondly, the associations between higher externalizing behavioral difficulties and learning difficulties (both math and reading) could be particularly strong in girls. Future studies will indicate whether girls could benefit the most from the treatment of both difficulties.

The results of this study, although with the limitations outlined above, may be a springboard for projects aimed at raising awareness in parents for early signs of both academic and behavioral difficulties so as to implement timely and tailored interventions [50].

## Figures and Tables

**Table 1:** Pearson’s r correlations between the reading, arithmetic and behavioral measures.

Measures	1	2	3	4	5	6	7	8
**1. Reading comprehension**		0.124 **	−0.415	−0.286 **	−0.178	−0.229 **	−0.384 **	−0.226
**2. Reading speed**		-	−0.395 **	0.088	−0.064	−0.111	0.027 **	−0.051
**3. Reading accuracy**			-	0.274 **	0.237 **	0.255 **	0.362	0.237 **
**4. Mental arithmetic difficulties**				-	0.080	0.156 *	0.245 **	0.115 **
**5. Emotional difficulties**					-	0.294	0.385 **	0.400 **
**6. Conduct problems**						-	0.647 **	0.527 **
**7. Hyperactivity**							-	0.460 **
**8. Difficulties with peers**								-
**Mean**	8.427	1.948	3.496	0.769	1.567	1.145	2.741	0.957
**Standard Deviation**	1.828	1.722	3.633	1.120	1.934	1.792	2.937	1.488

* *p* < 0.05. ** *p* < 0.01.

**Table 2:** Sex differences on all the measures of interest.

Dependent Variable	Male M (SD)	Female M (SD)
Conduct problems	1.453 (1.887) ^a^	0.762 (1.546) ^b^
Hyperactivity	3.232 (3.043) ^a^	1.991 (2.591) ^b^
Difficulties with peers	1.145 (1.654) ^a^	0.754 (1.286) ^b^
Emotional difficulties	1.481 (1.925) ^a^	1.501 (1.876) ^a^
Reading comprehension	8.445 (1.791) ^a^	8.456 (1.832) ^a^
Reading speed	1.926 (0.801) ^a^	1.998 (2.512) ^a^
Reading accuracy (no. of errors)	3.245 (3.289) ^a^	3.376 (3.501) ^a^
Speed in mental arithmetic calculation	30.957 (19.123) ^a^	35.198 (20.978) ^b^
Accuracy in mental arithmetic calculation (no. of errors)	0.721 (1.085) ^a^	0.892 (1.212) ^a^

Note: Different subscripts indicate significantly different means (*p* < 0.05). Comparisons were run on raw scores.

**Table 3:** Mixed models predicting academic skills from indicators of emotional-behavioral difficulties. (Part 1).

	Reading Comprehension					
**Fixed Effects**	b	SE	df	t	LLCI	ULCI
Intercept	−0.026	0.081	17.521	−0.327	−0.198	0.145
Sex	−0.183 *	0.091	430.463	−2.005 *	−0.364	−0.003
Conduct problems	0.023	0.079	433.894	0.293	−0.132	0.178
Hyperactivity	−0.364 ***	0.071	435.062	−5.127 ***	−0.504	−0.225
Difficulties with peers	−0.145 *	0.071	426.748	−2.042 *	−0.286	−0.005
Emotional difficulties	−0.126 *	0.060	434.678	−2.081 *	−0.246	−0.007
Conduct problems X Sex	−0.026	0.157	431.956	−0.167	−0.335	0.282
Hyperactivity X Sex	0.022	0.139	428.542	0.164	−0.251	0.297
Difficulties with peers X Sex	−0.235 ^†^	0.134	423.444	−1.750 ^†^	−0.499	0.028
Emotional difficulties X Sex	0.017	0.116	427.573	0.150	−0.210	0.245
**Random Effects**	b			Z Wald		
Σu	0.088 *			1.990 *		
Σe	0.816 ***			14.442 ***		
Deviance						
−2LL (df)	1224.858 (12)					
AIC	1228.858					

^†^*p* < 0.10. * *p* < 0.05. *** *p* < 0.001. Note: Sex: male −0.5 and female 0.5; such values allow to interpret the intercept value as referring to a generic child, with no sex assigned. LLCI, lower-level confidence interval (95%); ULCI, upper-level confidence interval (95%). σu: estimate of the residual error variance. σe: estimate of the intercept variance. −2LL: −2 Log likelihood. AIC: Akaike’s Information Criterion.

**Table 4:** Mixed models predicting academic skills from indicators of emotional-behavioral difficulties. (Part 2).

	Reading Speed					
**Fixed Effects**	B	SE	df	T	LLCI	ULCI
Intercept	−0.048	0.035	20.302	−1.379	−0.121	0.024
Sex	−0.097 *	0.043	422.509	−2.236 *	−0.183	−0.011
Conduct problems	−0.007	0.038	424.244	−0.209	−0.083	0.067
Hyperactivity	−0.120 ***	0.033	426.456	−3.551 ***	−0.187	−0.053
Difficulties with peers	0.018	0.034	413.404	0.535	−0.048	0.085
Emotional difficulties	−0.016	0.029	420.211	−0.567	−0.073	0.040
Conduct problems X Sex	0.032	0.076	423.930	0.423	−0.117	0.182
Hyperactivity X Sex	−0.044	0.066	421.226	−0.663	−0.175	0.086
Difficulties with peers X Sex	−0.008	0.064	415.374	−0.125	−0.135	0.119
Emotional difficulties X Sex	−0.087	0.056	421.699	−1.564	−0.197	0.022
**Random Effects**	b			Z Wald		
Σu	0.015 ^†^			1.931 ^†^		
Σe	0.181 ***			14.302 ***		
Deviance						
−2LL (df)	558.268(12)					
AIC	552.268					

^†^*p* < 0.10. * *p* < 0.05. *** *p* < 0.001. Note: Sex: male −0.5 and female 0.5; such values allow interpreting the intercept value as referring to a generic child, with no sex assigned. LLCI, lower-level confidence interval (95%); ULCI, upper-level confidence interval (95%). σu: estimate of the residual error variance. σe: estimate of the intercept variance. −2LL: −2 Log likelihood. AIC: Akaike’s Information Criterion.

**Table 5:** Mixed models predicting academic skills from indicators of emotional-behavioral difficulties (Part 3).

	Reading Accuracy (No. of Errors)					
**Fixed Effects**	b	SE	df	T	LLCI	ULCI
Intercept	0.084	0.105	18.815	0.801	−0.136	0.306
Sex	0.182 *	0.081	416.827	2.236 *	0.022	0.342
Conduct problems	−0.065	0.071	419.604	−0.918	−0.204	0.074
Hyperactivity	0.408 ***	0.063	419.574	6.455 ***	0.284	0.532
Difficulties with peers	0.027	0.064	429.857	0.432	−0.099	0.154
Emotional difficulties	0.165 **	0.054	428.551	3.029 **	0.058	0.273
Conduct problems X Sex	−0.014	0.140	418.766	−0.103	−0.291	0.262
Hyperactivity X Sex	0.291 *	0.124	416.710	2.347 *	0.047	0.535
Difficulties with peers X Sex	−0.022	0.118	413.132	−0.190	−0.256	0.210
Emotional difficulties X Sex	−0.005	0.102	415.223	−0.057	−0.207	0.195
**Random Effects**	B			Z Wald		
Σu	0.188 *			2.589		
Σe	0.625 ***			14.328 ***		
Deviance						
−2LL (df)	1113.592 (12)					
AIC	1107.592					

* *p* < 0.05. ** *p* < 0.01. *** *p* < 0.001. Note. Sex: male −0.5 and female 0.5; such values allow interpreting the intercept value as referring to a generic child, with no sex assigned. LLCI, lower-level confidence interval (95%); ULCI, upper-level confidence interval (95%). σu: estimate of the residual error variance. σe: estimate of the intercept variance. −2LL: −2 Log likelihood. AIC: Akaike’s Information Criterion.

**Table 6:** Mixed models predicting academic skills from indicators of emotional-behavioral difficulties (Part 4).

	Mental Arithmetic Difficulties					
**Fixed Effects**	b	SE	Df	T	LLCI	ULCI
Intercept	0.064	0.096	16.810	0.667	−0.139	0.268
Sex	0.378 ***	0.090	390.936	4.179 ***	0.200	0.557
Conduct problems	0.023	0.078	394.176	0.302	−0.130	0.177
Hyperactivity	0.459 ***	0.070	396.608	6.489 ***	0.320	0.598
Difficulties with peers	−0.034	0.070	399.414	−0.494	−0.173	0.103
Emotional difficulties	0.091	0.060	400.601	1.496	−0.028	0.210
Conduct problems X Sex	0.368 *	0.154	392.529	2.383 *	0.064	0.673
Hyperactivity X Sex	0.179	0.137	388.996	1.306	−0.090	0.449
Difficulties with peers X Sex	−0.160	0.130	385.476	−0.226	−0.418	0.096
Emotional difficulties X Sex	−0.053	0.114	389.004	−0.471	−0.278	0.170
**Random Effects**	B			Z Wald		
Σu	0.134 *			2.215 *		
Σe	0.721 ***			13.811 ***		
Deviance						
−2LL (df)	1081.838 (12)					
AIC	1085.838					

* *p* < 0.05. *** *p* < 0.001. Note: Sex: male −0.5 and female 0.5; such values allow interpreting the intercept value as referring to a generic child, with no sex assigned. LLCI, lower-level confidence interval (95%); ULCI, upper-level confidence interval (95%). σu: estimate of the residual error variance. σe: estimate of the intercept variance. −2LL: −2 Log likelihood. AIC: Akaike’s Information Criterion.

**Table 7:** Results for the single slope analyses for exploring the significant interaction effect between hyperactivity and sex in the prediction of reading accuracy (no. of errors).

	Boys					
**Fixed Effects**	b	SE	df	t	LLCI	ULCI
Intercept	−0.029	0.103	19.348	−0.286	−0.246	0.187
Hyperactivity	0.293 ***	0.058	240.627	4.994 ***	0.177	0.409
**Random Effects**	B			Z Wald		
Σu	0.159 *			2.313 *		
Σe	0.607 ***			10.664 ***		
Deviance						
−2LL (df)	612.737 (4)					
AIC	616.737					
	**Girls**					
**Fixed Effects**	B	SE	df	t	LLCI	ULCI
Intercept	0.168	0.116	17.615	1.448	−0.076	0.412
Hyperactivity	0.589 ***	0.076	209.102	7.725 ***	0.438	0.739
**Random Effects**	B			Z Wald		
Σu	0.199 *			2.203 *		
Σe	0.637 ***			9.803 ***		
Deviance						
−2LL (df)	545.510 (4)					
AIC	549.510					

* *p* < 0.05. *** *p* < 0.001.

**Table 8:** Results for the single slope analyses for exploring the significant interaction effect between conduct problems and sex in the prediction of mental arithmetic difficulties.

	Boys					
**Fixed Effects**	B	SE	df	t	LLCI	ULCI
Intercept	−0.112	0.096	17.257	−1.165	−0.315	0.090
Conduct problems	0.123	0.072	230.000	1.705	−0.019	0.265
**Random Effects**	B			Z Wald		
Σu	0.098			1.684		
Σe	0.781 ***			10.292		
Deviance						
−2LL (df)	622.161 (4)					
AIC	626.161					
	**Girls**					
**Fixed Effects**	B	SE	df	t	LLCI	ULCI
Intercept	0.196	0.100	13.767	1.951	−0.019	0.413
Conduct problems	0.521 ***	0.098	185.863	5.317 ***	0.327	0.715
**Random Effects**	B			Z Wald		
Σu	0.098			1.366		
Σe	0.826 ***			9.064 ***		
Deviance						
−2LL (df)	515.428 (4)					
AIC	519.428					

*** *p* < 0.001.
